# Invagination intestinale aiguë révélant un lymphome T digestif chez l'adulte: à propos d'un cas

**DOI:** 10.11604/pamj.2019.33.153.18758

**Published:** 2019-07-01

**Authors:** Hamza Hasnaoui, Hicham El Bouhaddouti, Ouadii Mouaqit, El Bachir Benjelloun, Abdelmalek Ousadden, Khalid Ait Taleb

**Affiliations:** 1 Service de Chirurgie Viscérale A, CHU Hassan II, Fès, Maroc; 2 Faculté de Médecine et de Pharmacie, Université Sidi Mohamed Ben Abdellah, Fès, Maroc

**Keywords:** Invagination intestinale aiguë, cause organique, lymphome T digestif, Acute intestinal intussusception, organic cause, intestinal T-cell lymphoma

## Abstract

L'invagination intestinale aiguë est une pathologie du nourrisson et du petit enfant. Sa survenue chez l'adulte est très inhabituelle. Elle est d'étiologie diverse. Dans l'immense majorité des cas, elle est secondaire à une tumeur qui peut être bénigne ou maligne. Un lymphome T digestif révélé par invagination intestinale est une entité très rare. Nous rapportons le cas d'un patient de 43 ans, admis aux urgences du centre hospitalier universitaire Hassan II de Fès, Maroc pour une occlusion intestinale. Le scanner abdominal a montré une invagination intestinale aiguë grêlo-grêlique sur un épaississement pariétal digestif de l'anse incarcérée. Le traitement était une résection chirurgicale carcinologique à ciel ouvert. L'étude anatomopathologique et immunohistochimique de la pièce opératoire a conclu à un lymphome anaplasique à grandes cellules de type T. Après la chirurgie, une chimiothérapie est indiquée dans le but d'améliorer le pronostic et d'éviter une éventuelle rechute. L'invagination intestinale est une affection rare chez l'adulte. Elle conduit le plus souvent à la découverte d'une cause organique pouvant être tumorale. A partir de ce nouveau cas et après analyse de la littérature, nous discutons les caractéristiques cliniques, diagnostiques et les possibilités thérapeutiques de cette pathologie rare.

## Abstract (en)

Acute intestinal intussusception occurs primarily in infants and young children. It uncommonly occurs in adults. It can have various causes. In the vast majority of cases, it is secondary to a benign or malignant tumor. Intestinal T-cell lymphoma revealed by intestinal intussusception is very rare. We report the case of a 43-year old patient admitted with intestinal occlusion to the Emergency Department at the University Hospital Hassan II, Fes, Morocco. Abdominal CT scan showed acute intestinal intussusception associated with incarcerated bowel loop with bowel wall thickening. Treatment was based on open carcinological resection. Anatomopathological and immunohistochemical examination of the surgical specimen showed large T-cell anaplastic lymphoma. After surgery, chemotherapy was indicated to improve prognosis and to avoid a potential relapse. Intestinal intussusception rarely occurs in adults. It most often leads to the detection of an organic cause such as a tumor. This study and literature review aim to highlight the clinical and diagnostic features as well as the therapeutic approaches for this rare disease.

## Introduction

L'invagination intestinale aiguë (IIA) de l'adulte, à la différence de l'enfant, est une manifestation rare survenant le plus souvent au cours d'une tumeur de la grêle d'origine maligne. Elle représente 1 à 5% des étiologies d'occlusion intestinale chez l'adulte [1]. Son mode évolutif est habituellement chronique ou subaigu [2,3]. Elle est rarement découverte devant un tableau aigu d'occlusion intestinale ou de péritonite [4]. Chez l'adulte une cause organique est trouvée dans 70 à 90% des cas, alors que, chez l'enfant l'invagination intestinale est le plus souvent idiopathique [2,5]. En conséquence chez l'adulte, le traitement est chirurgical fondé sur la résection intestinale avec cependant un débat encore ouvert concernant la nécessité ou non d'une réduction préalable du boudin d'invagination [1,5]. Nous rapportons un cas rare d'invagination intestinale aiguë révélant un lymphome T digestif chez un homme de 43 ans admis aux urgences dans un tableau d'occlusion.

## Patient et observation

**Figure 1:**
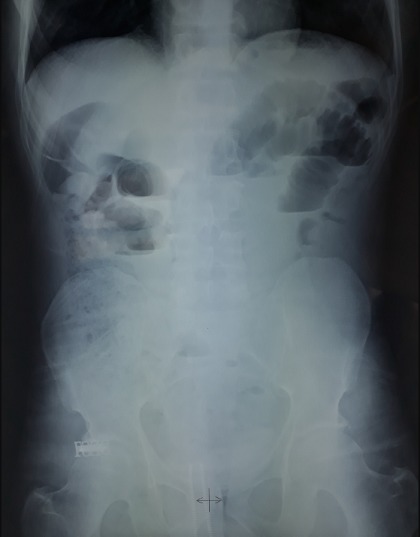
ASP en position debout montrant des niveaux hydro-aériques de type grêlique

**Figure 2:**
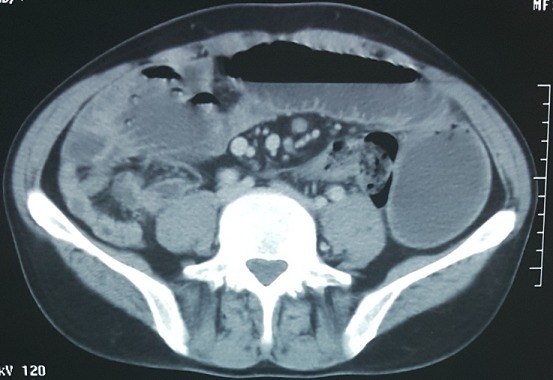
Image scannographique en coupe axiale montrant un syndrome occlusif en amont d'une invagination intestinale aiguë grêlo grêlique sur un épaississement pariétal digestif de l'anse incarcérée

**Figure 3:**
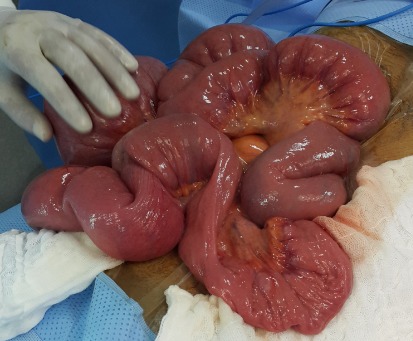
Image per opératoire montrant une invagination intestinale grêlo grêlique

**Figure 4:**
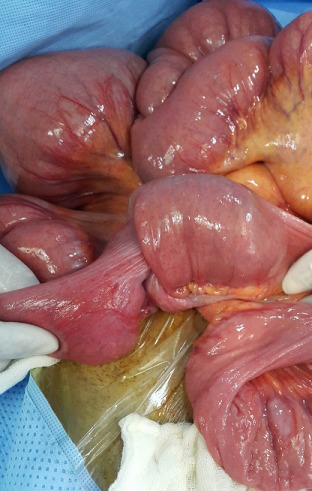
Image per opératoire montrant la tumeur grêlique responsable de l'invagination

**Figure 5:**
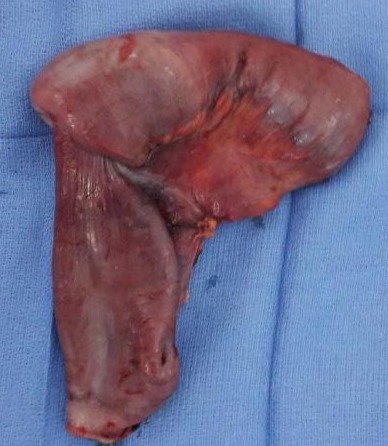
Image de la pièce opératoire (vue antérieure)

**Figure 6:**
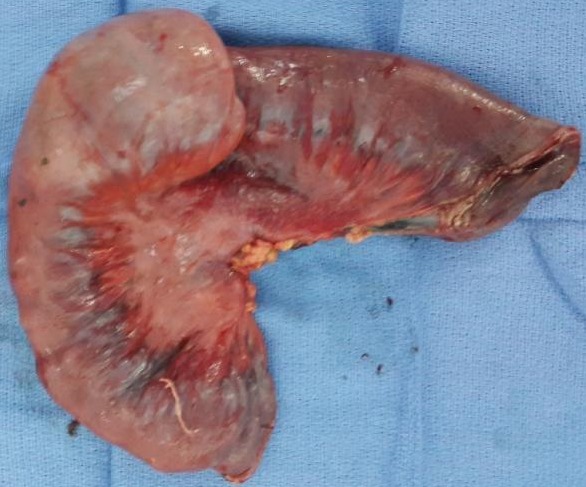
Image de la pièce opératoire (vue postérieure)

Un homme âgé de 43 ans, sans antécédents particuliers, admis aux urgences pour des douleurs abdominales diffuses avec notion d'arrêt des matières et des gaz et de vomissements bilieux. Le début de sa symptomatologie clinique remontait à un mois par la survenue de douleurs abdominales paroxystiques diffuses à type de crampes avec vomissements. Son transit s'était modifié avec une tendance à la constipation, parfois associée à des selles liquides. Ce syndrome abdominal a été résolutif puis entrecoupé d'épisodes douloureux paroxystiques jusqu'au jour de son hospitalisation motivée par l'accentuation des douleurs et l'arrêt des matières et des gaz. A l'admission, l'examen clinique objective un abdomen distendu avec tympanisme à la percussion, légèrement sensible, sans masse palpable, les orifices herniaires étaient libres. Le toucher rectal était normal. Le patient était apyrétique. Le reste de l'examen clinique était normal, mais il existait une altération récente de l'état général. Les examens biologiques usuels étaient sans particularité. La radiographie de l'abdomen sans préparation montrait des niveaux hydroaériques grêliques (Figure 1). Le scanner abdominopelvien montrait un syndrome occlusif en amont d'une invagination intestinale aiguë grêlo grêlique sur un épaississement pariétal digestif de l'anse incarcérée (Figure 2). L'indication opératoire était formelle. L'intervention chirurgicale, menée par une laparotomie médiane à cheval sur l'ombilic, a permis de confirmer que l'IIA est en rapport avec tumeur d'environ 8 cm de grand axe, située à environ 2m 20 de l'angle de Treitz et à 2m 80 de la valvule iléo-coecale (Figure 3, Figure 4), cette invagination était responsable d'une distension grêlique importante d'amont. Le boudin invaginé était viable. Présence également de plusieurs adénopathies au niveau mésentérique surtout au niveau de la racine du mésentère. Le geste a consisté sur une résection grêlique carcinologique emportant la tumeur et quelques ganglions mésentériques (Figure 5, Figure 6) puis anastomose grêlo grêlique termino terminale au même temps, et la réalisation d'une biopsie sur une adénopathie mésentérique. L'étude anatomopathologique et immunohistochimique de la pièce opératoire a conclu à un lymphome anaplasique à grandes cellules de type T (CD30+, ALK-). Les suites opératoires étaient simples et après 3 semaines, le patient était mis sous chimiothérapie adjuvante selon le protocole R-CHOP (Rituximab, Cyclophosphamide, Doxorubicine, Vincristine, Prednisone) avec une bonne tolérance et contrôle de la pathologie néoplasique.

## Discussion

L'invagination intestinale représente 1 à 5% des étiologies d'occlusions intestinales chez l'adulte, et 0,003 à 0,02% des hospitalisations ou une cause organique est trouvée dans 70 à 90% des cas et idiopathique dans 8 à 20% alors que, chez l'enfant l'invagination intestinale est primitive dans 90% cas [6,7]. La première invagination intestinale a été décrite par Barbette d'Amsterdam en 1674 [8] et Sir Jonathan Hutchinson qui a réalisé la première intervention chirurgicale d'invagination intestinale en 1871. Si cette affection ne s'observe que très rarement dans les pays développés, elle est au contraire relativement fréquente en Afrique et notamment en zones intertropicales. Les raisons de ces différences géographiques sont inconnues et certains facteurs tels que la diététique et les parasites sont évoqués [9]. Il est difficile de retrouver une prédominance liée au sexe ou à une tranche d'âge; même si l'âge moyen des différentes séries publiées se situe entre 40 et 50 ans avec des extrêmes allant de 15 ans à 81 ans [1,10,11]. La symptomatologie clinique est polymorphe et le plus souvent trompeuse: tableau occlusif aigu, tableau subocclusif de survenue progressive s'étendant de quelques jours à quelques semaines, syndromes abdominaux non spécifiques (modification du transit, douleurs abdominales diffuses, saignements digestifs), évoluant parfois pendant plusieurs mois, avec ou sans altération de l'état général [12,13]. La constatation à l'examen physique du malade d'une masse abdominale est un signe de grande valeur en particulier, si elle apparaît de siège et de consistance différents au cours d'examens répétés. Une fois sur deux en moyenne lorsqu'on est appelé à voir le malade en pleine crise, si le pannicule adipeux et le ballonnement abdominal ne sont pas trop importants, et si le relâchement musculaire de la paroi est suffisant, on sentira la tuméfaction correspondante au boudin d'invagination. On le cherchera soigneusement en décubitus latéral droit et gauche, en décubitus dorsal et en position de Trendelenburg [14,15].

Anatomiquement, l'iléon est considéré comme une zone d'atteinte préférentielle, les invaginations colo-coliques ne présentent que 27% des cas. Plus rares sont les invaginations colorectales, colo-anales ou jéjuno-gastriques [16]. Contrairement aux formes primitives du nourrisson. Une lésion organique est retrouvée au point de faiblesse de l'invagination dans 80% des cas chez l'adulte. Les tumeurs malignes représentent la première étiologie des invaginations chez l'adulte surtout au niveau du colon, alors qu'elles sont secondaires à une lésion bénigne (surtout au niveau du grêle) dans 25% des cas et 10% idiopathiques [17]. Ces lésions organiques sont représentées par les tumeurs stromales, les lipomes, les polypes, les adénopathies, les épaississements digestifs surtout iléocaecales. Le mélanome, l'adénocarcinome et les métastases sont retrouvés dans environ 15% des invaginations [18]. L'invagination intestinale aiguë sur un lymphome grêlique est rare, comme le cas de ce patient. Classiquement chez l'adulte, l'évolution de l'invagination est chronique avec des douleurs abdominales intermittentes associées à des crises sub-occlusives. La forme aiguë est surtout l'apanage des formes iléo-iléale. Pour Mondor, la forme aiguë serait le stade ultime d'une invagination chronique pour laquelle un diagnostic précoce n'aurait pas été fait [3]. C'est le cas de notre patient qui avait des douleurs paroxystiques depuis un mois précédant un syndrome subocclusif. Quelle que soit la présentation clinique initiale, le diagnostic se fait majoritairement par l'imagerie (échographie, scanner), plus rarement par la chirurgie exploratrice. Sur le plan radiologique, les radiographies de l'abdomen sans préparation peuvent contribuer à poser le diagnostic d'occlusion de l'intestin grêle, la visualisation directe de la tête du boudin sous forme d'une masse de tonalité hydrique moulée par de l'air du segment intestinal d'aval est très rare [1]; mais dans la plupart des cas, cet examen fournit peu de renseignements. Notre malade avait des niveaux hydro-aériques de type grêlique. L'échographie abdominale est un examen fiable et paraît prometteuse pour le diagnostic d'invagination intestinale [4,5], elle donne typiquement en coupe longitudinale une image en cible avec deux anneaux hypoéchogènes périphériques et un anneau central échogène, et en coupe transversale [4,5] une image en «sandwich» avec trois cylindres superposés, qui correspond au boudin d'invagination. L'échographie abdominale associée au doppler couleur peut dans certains cas mettre en évidence la disparition de l'hyperémie veineuse et artérielle du boudin d'invagination évocatrice de nécrose ischémique [19,20].

Malgré l'importance des données que fournis l'échographie, elle reste souvent gênée par la présence d'air en cas d'occlusion. Notre malade n'a pas bénéficié d'une échographie abdominale. Le scanner abdominal avec injection de produit de contraste, réalisé en urgence, permet d'augmenter la sensibilité du diagnostic qui peut atteindre 90% avec une spécificité de 100% chez l'adulte [21]. Il permet de diagnostiquer le syndrome obstructif, son mécanisme, en l'occurrence l'invagination, sa localisation précise et de montrer sa cause (masse intraluminale ou extraluminale). Il peut détecter une cause organique dans 71% des cas. Son rôle est plus important en cas de suspicion d'un lymphome abdominal, de lipome, de lésion tissulaire en rapport avec un polype. Il permet d'objectiver un épaississement de la paroi digestive associé à des adénopathies en cas de lymphome, une lésion intraluminale de densité graisseuse au centre entourée d'une paroi digestive en cas de lipome, ou de densité tissulaire en cas de polype. Les deux images classiques sont l'image «en sandwich» en coupe longitudinale dessinant la tête de l'IIA et l'image «en cocarde» en coupe transversale montrant le boudin de l'IIA. Dans notre cas, le scanner a été d'un grand apport; il a permis la mise en évidence d'un syndrome occlusif en amont d'une invagination intestinale aiguë grêlo grêlique sur un épaississement pariétal digestif de l'anse incarcérée avec plusieurs adénopathies coelio mésentériques faisant évoquer une origine tumorale. Le traitement est toujours chirurgical chez l'adulte et ne laisse aucune place à la réduction par hyperpression sous contrôle radiologique. Une résection plus ou moins étendue peut être nécessaire [22]. Le recours à une simple désinvagination est licite dans les formes idiopathiques. L'exérèse intestinale tout en respectant les impératifs carcinologiques s'impose lors de la découverte d'une tumeur à l'évidence maligne. Notre malade a bénéficié d'une résection grêlique carcinologique emportant la tumeur et quelques ganglions mésentériques puis anastomose grêlo grêlique termino terminale au même temps. L'étude anatomopathologique est nécessaire pour la confirmation diagnostique et doit être complétée dans certains cas par une étude immunohistochimique (le cas des lymphomes). Dans notre cas l'histologie et l'immunohistochimie ont conclu à un lymphome anaplasique à grandes cellules de type T (cd30+, alk-). Le pronostic est lié à la durée d'évolution, à l'étendue des lésions et à la nature de la cause [23].

## Conclusion

L'invagination intestinale chez l'adulte est souvent secondaire à une lésion organique: tumorale ou inflammatoire. Elle se caractérise par son polymorphisme clinique. Il s'agit essentiellement de phénomènes subocclusifs à répétition. L'échographie et surtout le scanner ont une place incontournable dans le diagnostic de l'invagination et de sa cause. Concernant le traitement de l'invagination intestinale de l'adulte, la résection du segment invaginé est toujours nécessaire car cet accident n'est qu'un épiphénomène à la base duquel se trouve dans 80% des cas une lésion organique qui doit être traitée.

## Conflits d’intérêts

Les auteurs ne déclarent aucun conflit d'intérêts.
